# Dynamics of the Negative Discourse Toward COVID-19 Vaccines: Topic Modeling Study and an Annotated Data Set of Twitter Posts

**DOI:** 10.2196/41319

**Published:** 2023-04-12

**Authors:** Gabriel Lindelöf, Talayeh Aledavood, Barbara Keller

**Affiliations:** 1 Department of Computer Science Aalto University Espoo Finland; 2 Department of Management and Engineering Linköping University Linköping Sweden

**Keywords:** COVID-19 vaccines, SARS-CoV-2, vaccine hesitancy, social media, Twitter, natural language processing, machine learning, stance detection, topic modeling

## Abstract

**Background:**

Since the onset of the COVID-19 pandemic, vaccines have been an important topic in public discourse. The discussions around vaccines are polarized, as some see them as an important measure to end the pandemic, and others are hesitant or find them harmful. A substantial portion of these discussions occurs openly on social media platforms. This allows us to closely monitor the opinions of different groups and their changes over time.

**Objective:**

This study investigated posts related to COVID-19 vaccines on Twitter (Twitter Inc) and focused on those that had a negative stance toward vaccines. It examined the evolution of the percentage of negative tweets over time. It also examined the different topics discussed in these tweets to understand the concerns and discussion points of those holding a negative stance toward the vaccines.

**Methods:**

A data set of 16,713,238 English tweets related to COVID-19 vaccines was collected, covering the period from March 1, 2020, to July 31, 2021. We used the scikit-learn Python library to apply a support vector machine classifier to identify the tweets with a negative stance toward COVID-19 vaccines. A total of 5163 tweets were used to train the classifier, of which a subset of 2484 tweets was manually annotated by us and made publicly available along with this paper. We used the BERTopic model to extract the topics discussed within the negative tweets and investigate them, including how they changed over time.

**Results:**

We showed that the negativity with respect to COVID-19 vaccines has decreased over time along with the vaccine rollouts. We identified 37 topics of discussion and presented their respective importance over time. We showed that popular topics not only consisted of conspiratorial discussions, such as 5G towers and microchips, but also contained legitimate concerns around vaccination safety and side effects as well as concerns about policies. The most prevalent topic among vaccine-hesitant tweets was related to the use of messenger RNA and fears about its speculated negative effects on our DNA.

**Conclusions:**

Hesitancy toward vaccines existed before the COVID-19 pandemic. However, given the dimension of and circumstances surrounding the COVID-19 pandemic, some new areas of hesitancy and negativity toward COVID-19 vaccines have arisen, for example, whether there has been enough time for them to be properly tested. There is also an unprecedented number of conspiracy theories associated with them. Our study shows that even unpopular opinions or conspiracy theories can become widespread when paired with a widely popular discussion topic such as COVID-19 vaccines. Understanding the concerns, the discussed topics, and how they change over time is essential for policy makers and public health authorities to provide better in-time information and policies to facilitate the vaccination of the population in future similar crises.

## Introduction

### Background

Since the emergence of the COVID-19 pandemic, vaccines against the SARS-CoV-2 virus have become a highly salient topic in public discourse. Although most people agree that the pandemic should end as soon as possible, the opinions on how and which mechanisms or policies can be adopted to get there differ greatly. As a main point of disagreement, many see vaccination (and revaccination) of the majority of the world’s population as the only way to bring the pandemic fully under control, whereas another group of people is hesitant about or completely opposed to the idea of getting vaccinated. The importance and sensitivity of the topic of vaccination lead to a large amount of discourse, commonly expressed on social media platforms, which are often highly polarized. Under circumstances such as social distancing and remote work, imposed during the pandemic, social media platforms have played an even more central role in people’s lives [1]. In these web-based social spheres, people openly discuss and share their opinions on vaccines with each other globally. A better understanding of how these conversations have developed over time and more insights into the topics discussed in them can help us better understand those hesitant to get vaccinated or those with low confidence in the surrounding processes.

Many attempts have been made to define the concept of vaccine hesitancy [2]. One such attempt was made by the *Strategic Advisory Group of Experts* working group on vaccine hesitancy, who proposed the following definition “Vaccine hesitancy refers to delay in acceptance or refusal of vaccines despite availability of vaccine services. Vaccine hesitancy is complex and context specific, varying across time, place and vaccines. It is influenced by factors such as complacency, convenience and confidence” [3]. A word commonly used in relation to hesitancy is confidence, which highlights the aspects of trust in vaccines and trust in the actors involved in the vaccination process, such as health care workers, researchers, governments, and pharmaceutical companies [2]. This study focused on tweets that expressed a negative stance toward COVID-19 vaccines. These included tweets that expressed a hesitancy toward taking available vaccines, a negative attitude toward policies promoting vaccination, or distrust in the actors involved in the vaccination process. We explored how much space these voices occupied on social media and what the most prevalent topics of discussion were.

A better understanding of individuals with low trust in vaccines or a hesitancy toward taking a vaccine can help shape interventions to improve trust. This not only will be important to bring the current COVID-19 pandemic fully under control but can also contribute valuable insights into how communication can be shaped in similar situations in the future. Although previous studies have examined the discourse around COVID-19 vaccines on Twitter (Twitter Inc) [4,5], this study is one of the firsts to cover the entire period from the World Health Organization (WHO) declaration of the pandemic until the summer of 2021. The study also provides temporal insights into how these discussions have changed over time.

Our main contributions were as follows:

1. A classifier capable of identifying tweets that express a negative stance toward COVID-19 vaccines.
2. A timeline of the development of the percentage of tweets expressing a negative stance toward COVID-19 vaccines for the first 18 months of the COVID-19 pandemic.
3. An overview of the major topics discussed by those with a negative stance during this time, their development, and the events that co-occurred with the changes in the discourse.
4. A data set of 2484 tweets manually labeled with their stance toward the COVID-19 vaccines, along with the codebook used in the annotation process [6].

### Sentiment Analysis

A number of approaches have been adopted to analyze the sentiments around infectious diseases on social media [7]. Sentiment mining on social media has proven to be a valuable resource for understanding people’s opinions about ongoing events and could potentially help with controlling pandemics [8]. Previous studies that investigated the sentiments around infectious diseases can broadly be grouped into 3 categories: lexicon based, machine learning based, and hybrids comprising the two [7]. Before the dawn of the COVID-19 pandemic, Du et al [9] used a machine learning approach to investigate the stance toward human papillomavirus (HPV) vaccines on Twitter. Using an annotated data set of 6000 tweets, they were able to train a support vector machine (SVM) classifier that could classify tweets as positive, negative, or neutral with satisfactory performance.

At the beginning of the COVID-19 pandemic, Medford et al [10] investigated the sentiments discussed on Twitter. They found that approximately 50% of COVID-19–related tweets could be classified as showing fear, whereas 30% could be classified as expressing surprise. Dominating topics discussed were the economic and political impacts of the virus, quarantine efforts, the transmission of the virus, and how to prevent it.

### Stance Detection

Stance detection analyzes textual input from people and identifies whether someone is favorable, against, or neutral toward a certain topic. Stance detection is related to sentiment analysis but is known to be a more difficult task [11]. Sentiment analysis aims to identify the opinion of a person and determine whether their textual input bears positive, negative, or neutral sentiments. A negative sentiment of a text does not always imply an unfavorable stance toward the topic. For example, Skeppstedt et al [11] used the phrase, “The diseases that vaccination can protect you from are horrible.” This phrase is favorable toward the prechosen topic of vaccination but contains negative sentiments toward an identified topic, which is *diseases*. However, in previous studies, there was not always a clear distinction between stance detection and sentiment analysis. For instance, some studies on the stance of social media users toward vaccination did not use the term stance [12]. Stance detection has also been used by Cotfas et al [13] to study hesitancy during the month following the UK vaccination rollout. Their cleaned data set contained approximately 1.2 million tweets, and approximately 7 out of 10 tweets were classified as having a neutral stance, and 2 out of 10 tweets were classified as having a negative stance. Some of the larger peaks in the volume of negative tweets correlated with the rollout of the Pfizer vaccine (Pfizer Inc; December 8, 2020), the US Food and Drug Administration approval of the Moderna vaccine (Moderna, Inc; December 17, 2020), and the vaccination dry run in India (January 2, 2021) [13]. Some of the most discussed topics were labeled as being about mistrust, scam, side effects, and the hiding of relevant information. The period before or after the month of the rollout was not studied, nor was there investigation about how the popularity of the found topics developed over time.

### Vaccination Discourse on Social Media

The widespread use of social media provides the opportunity to gain insights into the opinions of a broad population. A topic of particular interest with respect to public health is the discourse around vaccination. Understanding the concerns and insecurities about vaccinations can help policy makers find an appropriate way to address them. In 2011, Salathé and Khandelwal [14] assessed the sentiments expressed toward vaccination in tweets in the fall wave of the H1N1 (swine flu) pandemic. The authors could establish a correlation between the sentiments expressed on Twitter and the corresponding estimated vaccination rate (obtained via phone surveys) in the same area. By contrast, another study on the discourse on common flu vaccination on Facebook (Meta Platforms Inc) found an asymmetric participation of vaccination defenders and critics [15]. Although the defenders were able to reach 24% of the investigated network, the vaccination critics were only able to reach 8% of the investigated network.

With the onset of the COVID-19 pandemic, extensive work was done in the area of COVID-19 vaccinations. Some studies focused on the demographics of the vaccination-hesitant population [16,17], whereas others tried to understand what leads to this hesitancy [18,19], finding, similar to our study, that common concerns are potential side effects and distrust toward the pharmaceutical industry. Melton et al [20] used an approach similar to that of ours but focused on the social media platform Reddit (Reddit Inc). They investigated topics of vaccine discussion from December 1, 2020, to May 15, 2021, and found that a majority of posts used a positive tone. This is in line with our finding that posts classified as negative accounted for less than 10% of the posts in the data set throughout the investigated period. Lyu et al [21] focused on tweets about COVID-19 from the declaration of the pandemic until February 2021. Their results showed that opinions and emotions around vaccines were the most tweeted topics and that sentiments became more positive over time, similar to our finding that the percentage of negative tweets decreased over time. Several studies have used Twitter data to examine users’ sentiments and the discourse around vaccination and vaccine rollouts in different countries, such as Canada [22], South Korea [23], and Japan [24]. Similar to our study, the study by Chandrasekaran et al [25] did not restrict Twitter data collection to a specific country or region but focused on a 4-month period starting from January 1, 2021, a month after the first COVID-19 vaccination was already administered. They paid special attention to excluding company posts to ensure that they captured the attitudes of individuals and found that the average compound sentiment scores were negative throughout the investigated period [25].

Building on prior work on stance detection and vaccination discourse in social media, we applied dynamic topic modeling to investigate the evolution of negative discourse on Twitter around COVID-19 vaccines. We focused on the English-speaking Twittersphere as a whole and analyzed how the discussed topics are linked to the emerging global news and events related to COVID-19 vaccines over time.

## Methods

### Data Set

Tweets were collected using the academic research track of the Twitter application programming interface (API). Using the full-archive search, we were able to retroactively collect tweets from each day for the period of March 1, 2020, to July 31, 2021. We chose March 1, 2020, as our starting point, as it is the first day of the month in which COVID-19 was declared a pandemic by the WHO. As vaccines are typically seen as a potential solution in the case of spreading diseases, we aimed to also capture the anticipation and attitude toward future COVID-19 vaccines. This allowed us to obtain an overview of the evolution of the sentiments toward COVID-19 vaccines starting from when the pandemic was declared and vaccinations were a mere hope, covering the time from when the first vaccines were announced up to when the vaccines were rolled out in many countries. We queried tweets in the English language containing synonyms of the words *COVID-19* and *vaccine*, excluding any retweets. A Python script using Twarc2 [26] was used to send requests and collect results.

**Figure 1:**
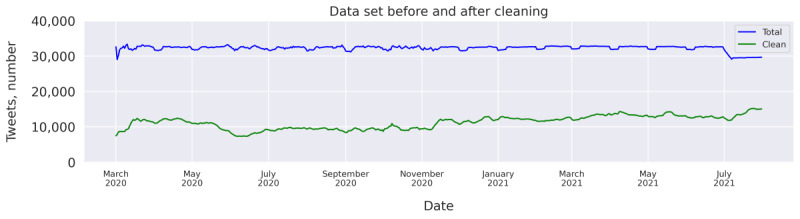
Seven-day moving average of tweets before and after cleaning.

Twitter does not provide a method for randomly sampling tweets when gathering historical tweets. Furthermore, the rate limiting of the API makes it difficult to efficiently collect tweets from a large number of time points per day. Therefore, tweets were collected at 6 different time points of each day, corresponding to noon in 6 major time zones with a large population of English speakers: AEST, IST, CET, EET, EST, and PST. This method makes it possible to collect a large number of tweets without hitting the rate limit while still covering high activity hours for different parts of the world. At each time point, 30 Twitter pages of tweets were collected, starting exactly at noon for each time zone. As pages do not necessarily correspond to a fixed number of tweets, there were some variations in the collected number of tweets from one day to another, as can be observed in Figure 1. The timespan of each collection also varied slightly based on how actively people tweeted immediately after noon and how long it took to fill the first 30 Twitter pages. For the first day of each month, as well as for all days in the month of July 2021, the API unexpectedly returned a lower number of tweets per page. Given the large number of collected tweets for each day, we did not consider this an issue for the analysis.

The collected data set contained a total of 16,713,238 tweets, with an average of 32,203 (SD 1458) tweets for each day in the period. To put these numbers into perspective, we also investigated how many tweets matched our search query in total. Using the *Counts Endpoint* of the Twitter API, we can quickly retrieve the number of tweets that match our keywords without having to go through the lengthy process of collecting them all. A total of 85 million tweets made during the investigated period matched the query, meaning that our sample represents approximately 20% of the relevant tweets.

As we were interested in the discourse led by people, tweets posted by the bots active on Twitter needed to be excluded. To investigate their prevalence in the data set, a random sample of 300 tweets was run through the OSoMe Botometer API (Osome Ltd) [27]. The botometer returns a *complete automation probability* that indicates the likelihood of an account being automated. Overall, 26% of the tweets were made from accounts with a complete automation probability >0.80, and of these accounts, 87% were sharing links. Shared links pose their own challenges in classification tasks. On the one hand, it is difficult to judge whether the shared link indeed reflects the stance of the sharer, and on the other hand, retrieving the information in the linked location can be difficult, especially as the information can change over time. On the basis of these challenges and the fact that many bots are sharing links, we decided to remove all tweets that contained links. Although we were aware that this removal would also exclude legitimate tweets made by humans, we expected that it would lead to a cleaner data set and a better classifier and introduce less overall bias. After this, 7,292,705 tweets remained, corresponding to 44% of the data set. Tweets with text content identical to the text content of other tweets from the same author were also removed to reduce the amount of spam, resulting in a total of 5,966,905 tweets. Finally, to reduce the overrepresentation of individual users, tweets made by users with >1000 tweets in this data set were removed. An example of such cases were bots that continuously announced free vaccination appointments. This removed 108,749 tweets, making the total number of tweets in the cleaned data set 5,858,156.

Google Trends is a website developed by Google that allows users to investigate how frequently a certain term was searched using Google’s search engine for a chosen period. It presents the user with the volume of the searched queries in different regions and languages over time. We gathered Google Trends data for the query “COVID passport” during the time of our data collection to compare the general search trend for this query with the popularity of this topic in our model. Google allows these data to be downloaded in CSV format directly from the website.

### Ethical Considerations

Twitter posts in our data set were made publicly available by the users and accessible at the time of data collection. In the annotated data set that we published, we only included the tweet ID's and did not include the posts. This means that if at a later point in time the users decided to delete their posts or make them unavailable, those posts will not be accessible to the future users of our data set. We present our analysis in a form that does not reveal the identities of the individuals who created the tweets. Consequently, the study did not meet the criteria for human subjects research and a review by an institutional review board was not required.

### Preprocessing

The text data of the collected tweets were extracted and transformed using a few common preprocessing steps. The text was lowercased, special characters were removed, and the “&” symbol was replaced with “and”; additionally, letters occurring more than twice in a row were removed (eg, helllo becomes hello). The text was also separated into units of words using the Natural Language Toolkit TweetTokenizer. Stop words contributing little meaning to the text (eg, *the*, *a*, and *in*) were removed using the Natural Language Toolkit English stop word list. As the terms used in the Twitter search query occur at least once in every tweet, they add bias and contribute only little additional information; therefore, they were also removed. The same preprocessing steps were performed on the text data for both the classifier and topic model. Preprocessing is not generally recommended for BERTopic unless the data contain a lot of noise such as HTML tags. However, in this case, preprocessing led to a better topic model. Perhaps the large volume of data analyzed in this study made the small loss of information due to the cleaning process negligible.

### Annotation

The first step to computationally categorizing documents (in our case tweets) into different groups (eg, negative and not negative stance toward COVID-19 vaccines) is to manually annotate a subsample of the documents with the correct labels as determined by the annotators. A codebook is often used to have a common framework among different annotators. The codebook sets out the rules that help decide which label a tweet receives. Although the classification in this study was limited to a binary negative or not negative labeling, we used 3 classes for our codebook. This made the annotation more intuitive and enables future studies to make a distinction between the neural and positive stances when required. This data set, which was annotated using the codebook in Table 1, is published along with this paper so that other researchers can benefit from it. In our codebook, a tweet can have a positive, negative, or neutral and unclear stance toward COVID-19 vaccines. The explanations of these categories were inspired by a previous codebook that classified tweets about HPV vaccines [9].

The tweets to be annotated were randomly sampled from the cleaned data set. The first 485 tweets were coded by the authors while the codebook was still being refined. Thereafter, 2 university students were hired to annotate 1999 more tweets using the developed codebook. Out of these, 21.86% (543/2484) of tweets were labeled positive, 19.77% (491/2484) were labeled negative, and the remaining 58.37% (1450/2484) were labeled neutral and unclear. The κ score (which quantifies the interannotator reliability) for the 3 categories was calculated to be 57.49, indicating moderate agreement. As the 3 categories could be considered ordered with neutral and unclear being an in-between of positive and negative, a weighted κ can also be of interest. The weighted κ score was calculated to be 0.605, indicating substantial agreement. All interannotator disagreements were then discussed in an attempt to find an agreement on the final label. If no agreement was reached, the label was set to neutral and unclear. Most disagreements were between neutral and unclear and the 2 other labels. There were only 22 disagreements where 1 annotator said positive and the other said negative.

**Table 1:** The codebook developed for the purpose of this study with example tweets for each category.

Stance	Definition	Example tweet
Positive	Showing positive opinion toward COVID-19 vaccines Prompting the uptake of the vaccines Expressing the intention of taking or having taken the vaccine	“Im getting vaccinated tomorrow yay”
Negative	Expressing concerns around safety, efficacy, injuries, or cost or resistance to COVID-19 vaccines due to cultural or emotional matters Discouraging the uptake of the vaccines Expressing the intention of not taking or refusing the vaccines Questioning the motives behind vaccine deployment, for example, those of scientists, pharmaceutical companies, or governments	“No mask and no COVID19 vaccine for me!”
Neutral	Contains no stance or the stance is unclear Expresses both pro and antistance at the same time Expresses the stance of someone else without own added input Not related to the COVID-19 vaccine topic Discussion on other medical treatments without relating it to vaccines Discussion on vaccines for other diseases Unclear what the person is trying to say	“Would you take a COVID vaccine?”

### Classifier

To investigate the discourse of those with a negative stance toward the vaccines, we had to find a way to identify tweets belonging to this category. Considering the size of the data set, we opted for a machine learning approach. Although there are many viable classification algorithms available, previous research classifying the stance toward vaccines on Twitter has shown success using SVMs [4,9,13]. Initial exploration comparing multinomial naive Bayes, random tree, and an SVM classifier also showed the most promising results for the SVM, leading us to choose this as our classification method.

SVM is a type of classification algorithm that can be used to automatically divide documents into categories [28]. In this study, each tweet was a document that needed to be labeled with either a *negative* or *not negative* stance. Using thousands of tweets annotated by hand as examples (training data set), the SVM learned to automatically label the remaining millions of tweets. The preprocessed tweets were vectorized using *term frequency–inverse document frequency*, giving each word a weight indicating how important it is in the text. The tweets that had been manually annotated were used to train the SVM to recognize tweets with a negative stance. The training data consisted of 2484 tweets annotated for the purpose of this study and 2679 annotated tweets made available by Cotfas et al [13]. In total, 23.77% (1227/5163) of the tweets in the data set were negative, whereas the remaining 76.23% (3936/5163) had the label *not negative*. Overall, 10.01% (517/5163) of the tweets were excluded from training and used to test the performance of the classifier. A randomized search with 3-fold cross-validation was used to find the optimized parameters for the vectorizer and SVM. As the focus was on negative tweets, it was a priority to minimize the number of tweets incorrectly labeled as belonging to this category.

### Topic Modeling

Topic modeling is a technique that is used to extract themes from a set of text documents. This study aimed to investigate discourse topics with a negative stance toward COVID-19 vaccines. Manually categorizing all 296,321 negative tweets into topics would have been incredibly time consuming, but topic modeling allows this to be done automatically in a matter of hours. We used BERTopic to train a model and label each tweet with a topic. BERTopic is a topic modeling technique that uses a complex language model (Bidirectional Encoder Representations from Transformers model) to cluster documents based on semantic similarity. For this study, we used the Python implementation of this model available as a package [29]. A Bidirectional Encoder Representations from Transformers model pretrained on a large set of texts can be fine-tuned to be used in a wide variety of language recognition tasks [30]. We used the BERTopic default embedding model, which was fine-tuned on 1 billion English sentence pairs.

The model was fitted on all tweets classified by the SVM as negative. BERTopic allows the user to specify the desired number of topics as well as the minimum number of documents that should constitute a topic. To keep the size of the model manageable, the minimum topic size was set to 500 documents, and the number of topics was set to “auto.” Words with a term frequency <0.0001 were also excluded. The resulting model contained 37 distinct topics. Although not every topic could be discussed within the scope of this paper, all 37 topics are reported in Table 2 for full transparency and reproducibility of the results.

To explore the changes in negative discourse over time, a dynamic topic model was also developed using BERTopic’s topics over time function. BERTopic has a parameter that controls the number of topic representations each topic should have in the timeline. A higher value gives more timestamps in the graph but risks decreasing the quality of the topic representations. This value was set to 35, as this number led to graphs with an appropriate level of granularity for our analysis. To allow for easier comparisons between topics of different sizes, the frequencies were normalized to values between 0 and 1. Therefore, if the peak of one topic is higher than that of another in the graphs, it should not be interpreted that the former topic is more popular than the latter topic; rather, the higher peak should be interpreted as the relative popularity for that particular topic.

**Table 2:** Topics of the model ordered by size.

Topic	Frequency^a^	Top words
UA^b,c^	103,953	gates, take, dont, bill
0	119,196	virus, take, flu, dont
1	10,415	dna, mrna, rna, gene
2	9431	pharma, trump, big, trust
3	9378	mask, masks, wear, wearing
4	3232	passport, passports, id, travel
5	3058	africa, africans, black, african
6	3037	children, kids, school, risk
7	2889	china, chinese, trust, virus
8	2820	guinea, pigs, pig, first
9	2159	exam, india, indian, oxygen
10	2048	pfizer, blood, clots, astrazeneca
11	1931	virus, coronaviruses, years, take
12	1787	5g, bill, gates, us
13	1749	bill, microchip, chip, tracking
14	1549	jab, jabs, experimental, jabbed
15	1363	hydroxychloroquine, zinc, gates, chloroquine
16	1289	test, testing, untested, rushed
17	1184	polio, measles, smallpox, pox
18	1089	russian, russia, putin, trust
19	1028	hiv, aids, years, 40
20	969	sars, sarscov2, years, mers
21	907	rushed, first, take, im
22	874	poison, poisonous, body, take
23	872	trust, dont, im, wouldnt
24	827	experimental, take, taking, experiment
25	794	antibodies, antibody, test, natural
26	772	boris, brexit, eu, borisjohnson
27^d^	737	username, username, username, username
28	729	liability, manufacturers, sue, liable
29	676	cure, treatment, want, cures
30	629	science, scientists, trust, dont
31	624	recovery, rate, 99, need
32	614	lockdown, lockdowns, want, dont
33	611	hcq, pharma, big, gates
34	570	injected, injection, inject, body
35	531	travel, fly, flying, airlines

^a^The frequency column contains the number of documents predicted to belong to each topic.

^b^UA: unassigned.

^c^The first topic UA contains unassigned documents that did not fit into any of the other topics.

^d^Topic 27 contained only usernames as top words, which are now censored.

## Results

In this section, we present the performance of the classifier and the findings based on the categorization of all the tweets in the clean data set using the classifier. We show how the negativity toward COVID-19 vaccines developed over the course of the pandemic, which topics made up this negative discourse, and how these individual topics evolved over time.

### Classifier Performance

The best classifier found using a randomized search was tested on 517 tweets that were not used in the training set; its performance is presented in Table 3. A macro *F*_1_-score of 0.67 was achieved with a precision of 0.8 on the negative class. This classifier used a radial basis function kernel and a vectorizer with 3000 features of unigrams and bigrams. The classifier was used to label the 5,858,156 tweets in the cleaned data set as having a negative or not negative stance toward COVID-19 vaccines. Of all the clean tweets, a total of 5.06% (296,321/5,858,156) of tweets were classified as having a negative stance.

**Table 3:** Performance of the support vector machine classifier.

	Precision	Recall	*F*_1_-score	Support
Negative	0.80	0.33	0.46	131
Other	0.81	0.97	0.88	386
Accuracy	N/A^a^	N/A	0.81	517
Macroaverage	0.80	0.65	0.67	517
Weighted average	0.81	0.81	0.78	517

^a^N/A: not applicable.

### Percentage of Negative Tweets Over Time

**Figure 2:**
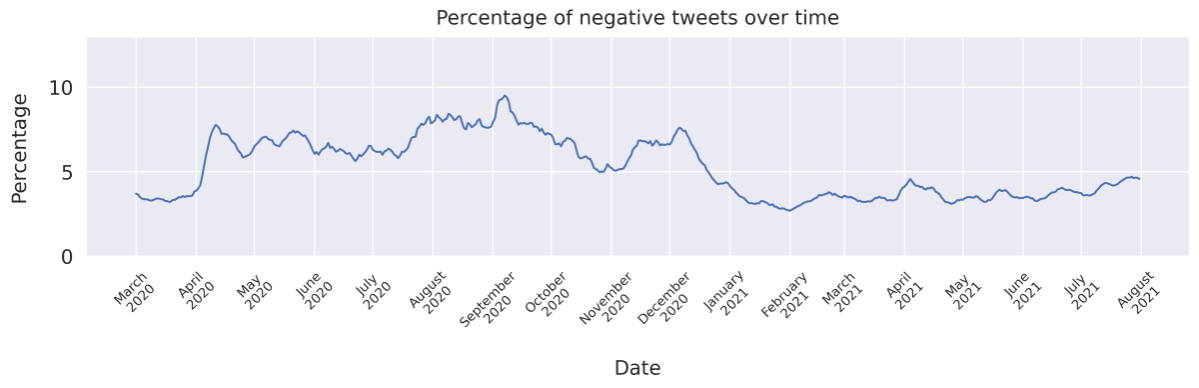
Seven-day moving average of the percentage of tweets classified as negative.

A timeline of the percentage of tweets with a negative stance toward COVID-19 vaccines during the period from March 1, 2020, to July 31, 2021, is shown in Figure 2. The average percentage of negativity was 5.1% (SD 1.9%). This number is slightly lower than that found by Cotfas et al [13] for the period from December 8, 2020, to January 7, 2021, where the percentage of tweets against COVID-19 vaccines was 6.78%. For the same period, our estimate was 4.7%. This discrepancy could likely be explained by the design choice of our study to use a conservative classifier that heavily prioritized not having false positives for the negative class, with the cost of more false negatives. Around the time of the WHO declaration of the pandemic (March 11, 2020), the amount of negativity was quite stable at 4%, and then, it rose in April 2020 and remained relatively high for the rest of the year. Co-occurring with the vaccination rollout in December was a decline in the share of negative tweets, and the following period showed similar negativity to that observed before the declaration of the pandemic.

### Topic Model of Negative Tweets

The produced topic model contained 37 different topics, with sizes ranging from 531 to 119,196 tweets. The topics are presented in Table 2 along with the top keywords that were calculated to be the most representative of each topic using the BERTopic’s modified version of term frequency–inverse document frequency. A dynamic topic model representing the popularity of each topic at 35 different time points in the investigated period was also created. In addition to the graphs included in the *Results* section, the timelines of all topics are available in an interactive form on the companion website [31].

**Figure 3:**
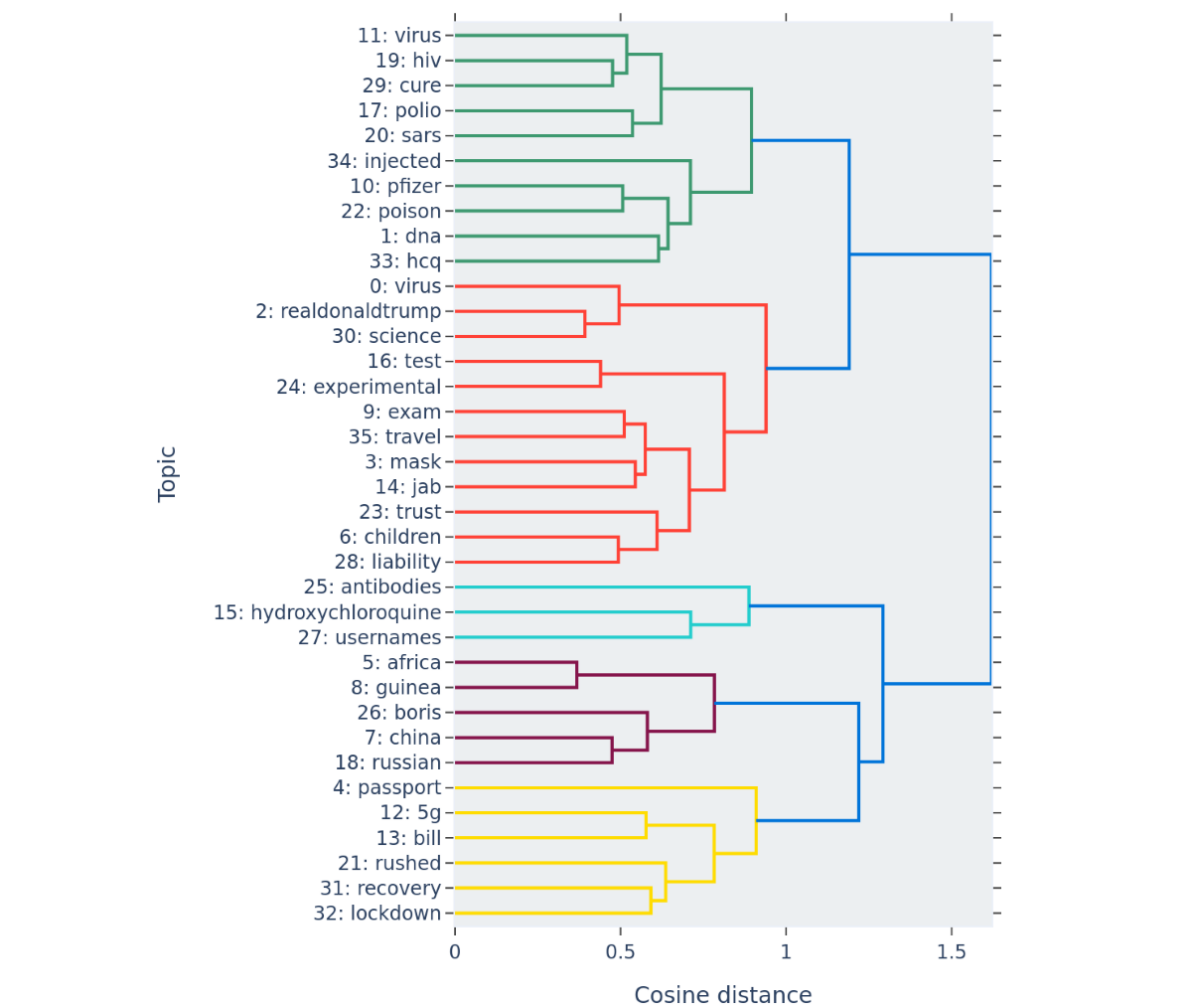
Hierarchical structure of the topics labeled with the topic index and the highest scoring word.

In this section, a selection of topics from the model is discussed using example tweets and graphs of their development over time. These topics are referred to with an index that can be used to locate them in Table 2. The dendrogram in Figure 3 shows how closely related the topics are according to our topic model. In the following discussion, we have chosen to group some topics together when discussing them; these groupings are based on their closeness in the dendrogram as well as qualitative similarities seen by the authors.

### Travel, COVID-19 Passports, and Territories (Topics 4, 7, 9, 18, and 35)

This group contained topics related to traveling and COVID-19 passports as well as discourse around the countries China, Russia, and India and the continent Africa.

Topic 4 is a cluster of documents discussing COVID-19 passports and certificates of different forms as a requirement for travel and other activities. The words “need” and “want” are highly scored in the representation, with many users arguing that they do not want or need a vaccine nor a passport. The word mandatory is also prevalent as a result of many discussing the enforcement of such passports.

**Figure 4:**
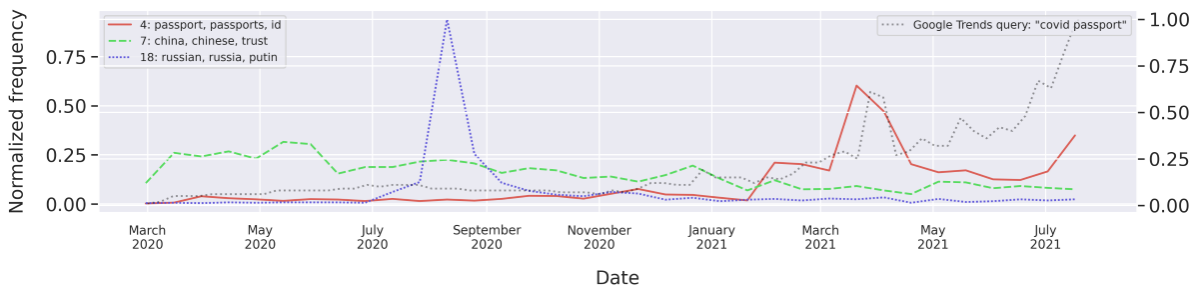
The normalized frequency of the “COVID-passports,” “China,” and “Russia” topics over time. For comparison, Google Trends normalized search frequency for “COVID passport” is shown (dashed gray line) using a separate axis to the right.

Figure 4 shows that this topic first gained popularity in the negative tweets by the beginning of 2021 as vaccines were being rolled out. This trend is also true for the general search interest, as indicated by the blue line showing the Google Trends popularity for the query “COVID passport.” The topic showed major peaks in both the topic model and Google Trends in May and July 2021. The first peak in April had an especially high search volume from Great Britain, as indicated when isolating this period in Google Trends. The go-ahead from Boris Johnson for COVID-19 passports could have likely been the event that sparked this discussion. Negative tweets from this period discussed COVID-19 passports in general, but many also spoke to Boris Johnson directly:

@BorisJohnson You must ensure GB does not push vaccine passports. It is intolerable to think Pharma and Gates Foundation should have any influence here. You are elected and accountable-they are not. The vaccinated can still pass on COVID. Stop penalising hard pressed medics to comply. Disgrace.

Topic 35 about travel contained a similar discussion but with a focus on travel, airlines, and going abroad and seemed to have had more constant popularity throughout the entire period.

The peak for July in Figure 4 is likely related to the European Union’s introduction of the EU Digital COVID Certificate. This was introduced to be used in travel within the Schengen area to prove vaccination, recovery, or recent negative test. Many tweets in this topic argued that the passports are unnecessary and unfair because vaccinated people can still transmit the virus. Others discussed the forging of COVID-19 passports:

The black market should have a fake vaccine passport or whatever they are calling it...This will be my approach

Anybody selling a fake vaccine passport yet? Or am I abit too early?

More conspiratorial voices also discussed how the vaccine passports were a part of a bigger plan:

No thanks. Vaccine passports where in plan before COVID-19.

China was also a major topic in the model, with 2889 negative tweets classified as belonging to this cluster. Major keywords for this topic were “chinese,” “trust,” and “fake.” Some tweets in this topic expressed distrust in China, even claiming that the virus was “manufactured” there:

I pondered why China has yet to come up with a vaccine for COVID since it began there. Then I remembered that any vaccine would be manufactured in China so why would they hurry? Why would they want to alert the WHO that this virus had to be taken seriously? China is a threat?

The topic saw an increase in popularity in mid-March 2020 (Figure 4), when the Chinese vaccine was approved for human testing. Some negative tweets from this period expressed an unwillingness to take a Chinese vaccine, whereas others discussed the conspiracies of a China-developed virus. This topic was highly salient in the negative discussion at the beginning of the pandemic and then slowly lost traction over time.

Russia was also a topic of negative discussion, with a majority of tweets between July and November 2020 and a major peak in August (Figure 4). This peak was likely due to the Russian vaccine Sputnik V being registered on August 11. Some of these tweets discussed whether one should trust a Russian vaccine:

I wouldn’t want a covid vaccine from putin would you?

It should be noted that our study only investigated tweets in English, and the topics about Russia and China are, therefore, mainly talking about these countries from a foreign perspective.

India is also a country prominent in the negative discussion, with a focus on their examinations. Most of the discussions in this topic concerned mandatory in-person examinations during the pandemic. It should be noted that the tweets on this topic may have posed a difficult challenge for the machine learning classifier; that is, some tweets that express negativity toward in-person examinations were incorrectly classified as having a negative stance toward the vaccines. For example, many tweets demanded a shift to web-based examinations until a vaccine has been made available:

Shift exams to online mode or no continuous exams. Everyone demanding for Delay in Compliance due dates. Think Same way, students are not punching bag for everyone. We are also human, we are not corona immune. We have not taken corona vaccine yet???

The topic about Africa contained several different themes of discussion. A major theme was the claim of racist motives behind the vaccines. Users argued that the vaccines were being sent to African countries as part of a secret plot by Bill Gates to test the side effects of the vaccines or for the purpose of population control. The words “bill,”, “guinea,” and “pigs” scored highly in the topic representation. Moreover, the topic also contained similar claims of racism against African Americans. This discourse seems to have been sparked by a statement by Melinda Gates where she proposed that the Black population should receive priority for the vaccines because they face disproportionate effects from the virus. Another theme of this topic was indicated by the keyword “madagascar” in the representation and concerned a tea used by the Madagascan population with claimed benefits against COVID-19, which some argued makes a vaccine redundant.

### Pharma and Alternatives to Vaccines (Topics 2, 15, 25, 28, 31, and 33)

Under this heading, we have grouped topics that concerned discussions around pharmaceutical companies, their profit motives, and proposed alternatives to vaccines. Topic 2 had “big,” “pharma,” “trust,” and “rushed” as highly scoring words, with many users questioning whether the vaccine was pushed primarily to make money:

Big Pharma can’t make as much money with a pill versus a year-long process for a vaccine.

Many users voiced their concerns about the vaccines directly to the then-sitting president, tagging “@realDonaldTrump” in their tweets. Another commonly raised critique in the discourse concerned the fact that vaccine manufacturers cannot be easily sued, which was seen in topic 28:

Who would want a vaccine when Drug companies can’t be sued if something goes wrong there has not been enough testing of vaccines to make sure they are safe.

Two topics (15 and 33) mostly discussed hydroxychloroquine as a better measure than the vaccines against COVID-19.

In topic 25, people discussed the protection antibodies give against the virus. A commonly raised argument was that the vaccines should not be necessary for those who were already infected by the virus. Topic 31, a topic with 624 tweets, was dominated by tweets citing a high recovery rate from COVID-19 as an argument against the vaccines. An example of a very typical tweet from this category is as follows:

If there is a 99% recovery rate, why would we need a vaccine?

**Figure 5:**
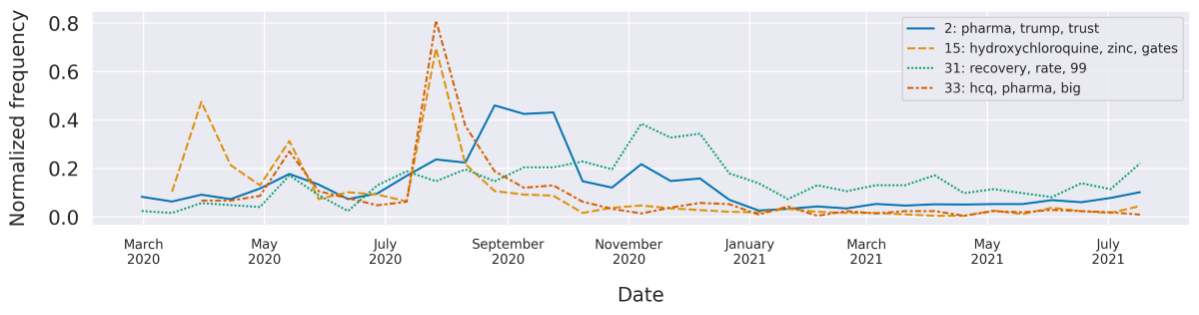
The normalized frequencies of the topics “hydroxychloroquine,” “pharma,” and “recovery” over time.

The topic grew in popularity during the second half of 2020, reaching a peak in the months leading up to the vaccine rollouts (Figure 5).

### Popular Conspiracies (Topics 12 and 13)

**Figure 6:**
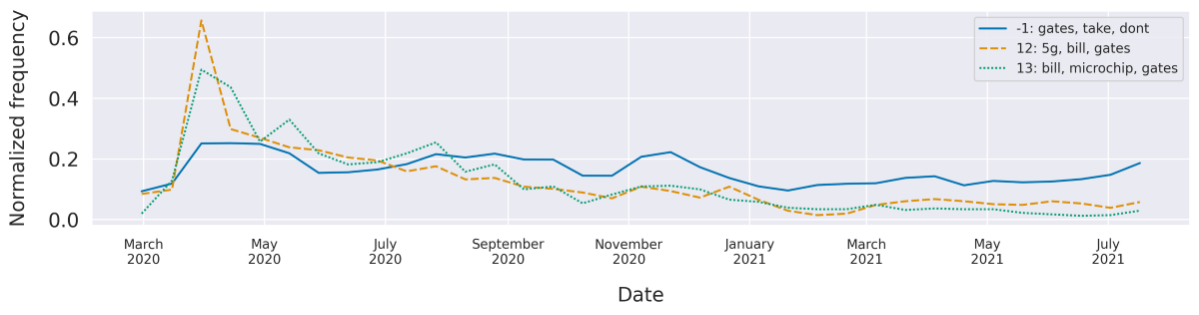
The normalized frequencies of the topics “Bill Gates,” “5G,” and “microchip” over time.

Topic 12 had a strong peak in April (Figure 6), which coincided with the time when the first 5G network tower was put on fire in Liverpool, United Kingdom (April 2) [32]. Apparently, a rumor started that 5G towers are partially to be blamed for the spread of COVID-19. This rumor was combined with the sentiment that the 5G towers are used to control people’s minds using microchips that are supposedly inserted through COVID-19 vaccination. The mayor of Liverpool declared this rumor as false, which seems to have triggered some people to take action into their own hands. This incident triggered discussions among the vaccine-hesitant population, as can be seen in our timeline, where the words “5G,” “tower,” “chips,” and “microchips” were highly weighted.

Topic 13 is closely related to the topic of 5G (topic 12) but with a stronger focus on the microchip rumor. The peak and curve behaviors are very similar for both topics (as they both share microchip as an important word). They peaked in the beginning of April and then lost their importance. The peak co-occurred with the following events, which may have contributed to it. On March 18, Bill Gates logged onto Reddit and answered questions. While doing so, he predicted that one day we would all carry a digital passport of our health records. He did not suggest a microchip for this but some kind of e-vaccine card. On March 19, a Swedish site picked this up and put the following headline to their article: “Bill Gates will use microchip implants to fight coronavirus.” With that, the conspiracy theory was born. The discussion it started among vaccine-hesitant people is reflected in our data. Simultaneously, the nongovernmental institution Digital Identity Alliance was brought into this, as they advocate for a digital ID for undocumented people such as refugees. Vaccine-hesitant individuals drew the conclusion that Digital Identity Alliance is involved in inserting microchips into people to reach their goal. Topic 13 had words such as “gates,” “implant,” and “tacking” as highly rated words, with many tweets talking about how the pandemic was planned by Gates:

It’s already planned by Gates. Thank Trump for not allowing ANY Gates vaccine!

The topic did not closely correlate with Google search volume for Gates, indicating that this could be a topic with a specific popularity on Twitter.

### Testing and Guinea Pigs (Topics 8 and 16)

This topic mostly contained tweets discussing the vaccine trials with a critical tone. The terms “pig” and “pigs” scored highly in this topic because of their appearance in the phrase “Guinea pigs,” which was used to refer to those choosing to take the vaccine. One of the users said the following:

I WILL NOT BE A GUINEA PIG with this vaccine! Why don’t you and your family be the test dummies!

The topic had the most popularity during the vaccine trials of 2020 and had since lost traction during the rollout of the vaccines.

Closely related is the topic of testing (topic 16), which also followed a similar trend over time. Important keywords for this topic such as “rushed,” “tested,” and “wouldn’t” reflected the stance that the development process is being “rushed” and an unwillingness to take an “untested” vaccine:

I have to agree with you on not taking it. This is an example of why vaccines should not be rushed with much needed testing not done before it’s approval.

### Pfizer and AstraZeneca (Topic 10)

**Figure 7:**
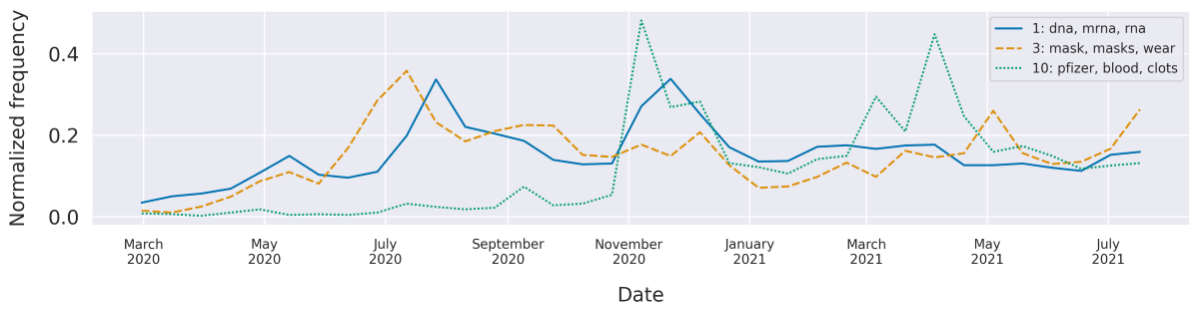
The normalized frequencies of the topics “Pfizer,” “mRNA,” and “mask” over time.

This topic peaked in November 2020 (Figure 7). Compared with Google Trends, there was a clear peak with the search terms for Pfizer and COVID-19 on November 9. This was the day on which Pfizer announced a successful phase 3 trial of their COVID-19 vaccine with an effectiveness of 90%. On the same day, “Nature” and “BBC News” reported on this. This announcement likely led to an increase in critical voices concerning the safety of the vaccine and triggered discussions among vaccine-hesitant groups. We also saw peaks of discourse during March 2021, with the key terms “aztrazeneca” and “blood clots” used especially often during this period. At this time, concerns surrounding a potential side effect of blood clots started circulating, causing many countries to pause their use of the AstraZeneca vaccine (AstraZeneca plc). These announcements seemed to have caused a lot of negative discourse around the vaccine, such as the following tweet about the corresponding user’s distrust in the process:

Denmark, Norway, Iceland and Bulgaria halt use of AstraZeneca’s COVID-19 vaccine over reports of blood clots. Has any research been done on effects on various blood types and regional DNA variances in countries?? Thought NOT. Don’t trust Chinese “run” WHO!

### DNA and Messenger RNA (Topic 1)

This topic had 2 peaks in the investigated timeline. The first peak was observed in August 2020 (Figure 7). Pfizer published the results of the phase 1 and phase 2 clinical trials for its messenger RNA (mRNA)-based vaccine in the journal *Nature* on August 12 [33]. Around this time, many news outlets started to describe promising vaccine candidates, with a special focus on the new technique of using mRNA, as was adopted by Pfizer and Moderna. This new technique of using the body’s own protein-generating mechanism to produce the spike protein of the SARS-CoV-2 instead of using a dead or weakened virus gave rise to concerns about the safety of the vaccines. Some people were concerned that it could have additional adverse effects on a human’s DNA and permanently alter it. The second peak of the mRNA topic in December 2020 lied close to a peak seen for the Pfizer topic, likely because of the publication of Pfizer’s phase 3 trials using the mRNA technique.

### Preventive Measures (Topics 3, 6, and 32)

One of the largest topics found in the data set with 8984 tweets concerned the use of face masks as a protection measure against the virus. The topic picked up pace in June 2020 (Figure 7) and remained popular throughout the study period. Top words for this topic included “mask,” “wearing,” “distancing,” “protect,” and “mandatory.” As the analysis was limited to tweets with a negative stance toward vaccines, much of the discourse consisted of critiques of mask mandates, which called them discriminatory. One of the users said the following:

Yet if I don’t take the covid ‘vaccine’ or wear a mask you want me to suffer total discrimination.

Other users expressed support for masks, seeing it as an alternative to vaccines:

Definitely masks but no to the vaccine. I don’t trust it because djt pushed them too fast.

In addition to masks, lockdowns are another measure that has been widely used in an attempt to limit the spread of the virus. Many users in topic 3 questioned policies being set in place to limit the spread of the virus. Tweets from early pandemic often argued that lockdowns are unfeasible with no end in sight for the pandemic. As the vaccines were deployed, this topic seemed to have shifted to the argument that the lockdowns have been put in place to persuade people to take what they perceive as an unnecessary vaccine.

The eighth largest topic of the model concerned the vaccination of children (topic 6). A commonly expressed view in this topic was that the vaccine is too experimental to use on children and that it is unethical to make vaccines mandatory for attending school. Many parents claimed that they will opt for homeschooling rather than a vaccine. An argument often used in this topic was that children have strong immune systems and should, therefore, not require a vaccine. Another common sentiment in this topic was that the fear of COVID-19 is overblown and that schools should be reopened.

### Parallels to Other Diseases (Topics 17, 19, and 20)

Three topics made comparisons between COVID-19 and other viral infections. The 3 most common diseases in our model were polio, HIV, and the disease caused by the earlier strains of SARS. Many tweets raised the argument that it is implausible that an effective vaccine has already been found for COVID-19, as many years of research never led to a successful vaccine for HIV or SARS. Many also questioned the mRNA technology, arguing that a vaccine developed using this technology is not a “real vaccine.” Some users bring up polio vaccines as an example of vaccines that actually worked because this disease has been eradicated in most places, something that cannot be said about COVID-19.

## Discussion

### Principal Findings

In this research, we investigated the negative discourse about COVID-19 vaccines that took place on Twitter in the English language between March 1, 2020, and July 31, 2021. Using the topic modeling methodology, we found 37 topics. We used dynamic topic modeling to show how the popularity of the topics changed over time. Our results indicate that the negative discourse about COVID-19 vaccines reduced along with the vaccine rollouts. This could suggest that the beneficial effects of the vaccines along with educational efforts have succeeded in reducing negative discussions, specifically the discourse involving conspiracy theories.

We developed a classifier to identify tweets with a negative stance toward vaccines and then modeled the topics of the negative tweets. Looking at the topics found, we see that a number of different conspiracy theories play a large role in the negative discourse. Some topics have an obvious link to popular conspiracy theories, such as those about 5G towers, microchips, and Bill Gates. However, in topics discussing other concerns such as COVID-19 passports, pharmaceutical companies, and racism, references to grand conspiracies are commonplace. Another overarching theme concerns negative perceptions of how restrictions and guidelines impact daily life. Distrust in pharmaceutical companies also seems to fuel much of the hesitancy and a discussion of alternatives to vaccines.

The percentage of negative tweets, as well as the more conspiratorial topics, saw a noteworthy increase in the month following the declaration of the pandemic. It appears that the focus placed on the pandemic gave fuel to these conspiracies, which previously held a more fringe position. Previous research has indicated that anxious people are more likely to believe in and share misinformation [34,35]. Therefore, it could be hypothesized that an increase in anxiety provoked by the news of a pandemic could explain the increase in hesitant and conspiratorial tweets in the following months. Furthermore, partaking in misinformation sharing has also been shown to further contribute to anxiety [36]. Therefore, it could be posed that a vicious circle could be formed where anxiousness leads people to share more misinformation, leading to even more anxiety. Another possible explanation is that these negative ideas were able to spread to a higher degree because the network of people involved in the discussion grew.

Similar to Lyu et al [21], we saw a general decline in the percentage of negative tweets co-occurring with the vaccine rollout. Furthermore, beyond the time frame investigated by Lyu et al [21], we could show that negativity stayed at this lower level during the following 7 months.

Decreased popularity was also observed for many of the more conspiratorial topics, such as those about 5G towers, microchips, and the Gates Foundation. This could indicate that communication by governments and health authorities around the issue was successful at combating some of the negative perceptions people had about the vaccines. However, another explanation could be that the deployment of vaccines attracted more public attention, shifting the composition of users involved in the discourse. Moreover, it cannot be ruled out that Twitter started enforcing its crisis misinformation policy more strictly as the vaccination process began, possibly contributing to a decrease in conspiratorial and vaccine-hesitant tweets.

### Limitations

It is important to note that this study is limited in its scope to only the English-speaking users of Twitter. Although Sloan et al [37] found that there is widespread Twitter use among the general population, the conversations observed in this study cannot be considered representative of the entirety of the general public discourse. Furthermore, the demographic using Twitter in English likely differs from the overall user base in many respects. Owing to the data collection limitations imposed by Twitter at the time, we were not able to collect all the relevant tweets for this work. We minimized the risk of missing prevalent topics by introducing the data collection strategy described in the section *Data Set* under *Methods*. The analysis of the topics was also limited to negative discourse. Future studies could benefit from investigating how the positive discourse has changed over the course of the pandemic. This could also help answer questions about which positive discussions took place as the vaccines rolled out, taking up the space previously held by negativity. As the pandemic has continued into 2022, new studies could also include an even longer period to obtain a more comprehensive picture of the evolution of topics over the course of the pandemic.

### Conclusions

Although the negativity toward vaccinations on Twitter decreased as the vaccines rolled out, many countries still faced difficulties with vaccination willingness among their populations. In our analysis of negative tweets over the course of the pandemic, we saw different indications for vaccine hesitation, such as low trust toward authorities, strong insistence on personal freedom with respect to following guidelines such as the wearing of masks or uptake of vaccines, and a nonnegligible influence of conspiracy theories around COVID-19 and vaccinations. Although the percentage of negative tweets and conspiratorial topics decreased as the vaccines rolled out, it is still possible that the negative discourse in the preceding months had already swayed some users into a more hesitant stance. As unvaccinated people face a much higher rate of hospitalization and death [38], hesitancy could have severely negative outcomes in the society. It has been estimated that in a country where nonpharmaceutical interventions are relaxed, hesitancy could lead to >7 times higher mortality [39]. To improve the handling of the current pandemic as well as prepare for future pandemics, a successful communication strategy should address concerns circulating on social media at an early stage, preventing negative perceptions from taking hold. The fact that bizarre conspiracies such as mind-controlling 5G antennas were widely circulated must be seen as a failure of communicative and educational efforts, and these conspiracies take up valuable space where constructive conversations around the pros and cons of vaccination could be had.

Our work and its methodology for examining the temporal evolution of the negative discourse around COVID-19 vaccines can be used to build tools for the near real-time monitoring of discussions around future health crises or similar events. For example, the Vaccine Sentimeter [40] was a tool designed for the real-time monitoring of the global view of vaccination conversations on the web. This tool was used to study conversations around polio and HPV vaccines. Our methodology would be more suitable for monitoring the changes in views over time in near real time, for example, comparing the change in topics from one week or month to the next. Such a tool can inform national and international health organizations, governments, and other stakeholders on how the public opinion toward a certain vaccine or even toward other global crises (eg, climate change) evolves over time.

## Abbreviations

API: application programming interface
HPV: human papillomavirus
mRNA: messenger RNA
SVM: support vector machine
WHO: World Health Organization

## Data Availability

The data set of tweets annotated for the purpose of this study has been made available on GitHub [6]. Plots can be viewed interactively on the companion website [31].
